# Deep Learning-Based Fusion of Optical, Radar, and LiDAR Data for Advancing Land Monitoring

**DOI:** 10.3390/s25164991

**Published:** 2025-08-12

**Authors:** Yizhe Li, Xinqing Xiao

**Affiliations:** College of Engineering, China Agricultural University, Beijing 100083, China; yizhe.li@cau.edu.cn

**Keywords:** land monitoring, synergistic harmonization, optical remote sensing, radar (SAR), machine learning

## Abstract

Accurate and timely land monitoring is crucial for addressing global environmental, economic, and societal challenges, including climate change, sustainable development, and disaster mitigation. While single-source remote sensing data offers significant capabilities, inherent limitations such as cloud cover interference (optical), speckle noise (radar), or limited spectral information (LiDAR) often hinder comprehensive and robust characterization of land surfaces. Recent advancements in synergistic harmonization technology for land monitoring, along with enhanced signal processing techniques and the integration of machine learning algorithms, have significantly broadened the scope and depth of geosciences. Therefore, it is essential to summarize the comprehensive applications of synergistic harmonization technology for geosciences, with a particular focus on recent advancements. Most of the existing review papers focus on the application of a single technology in a specific area, highlighting the need for a comprehensive review that integrates synergistic harmonization technology. This review provides a comprehensive review of advancements in land monitoring achieved through the synergistic harmonization of optical, radar, and LiDAR satellite technologies. It details the unique strengths and weaknesses of each sensor type, highlighting how their integration overcomes individual limitations by leveraging complementary information. This review analyzes current data harmonization and preprocessing techniques, various data fusion levels, and the transformative role of machine learning and deep learning algorithms, including emerging foundation models. Key applications across diverse domains such as land cover/land use mapping, change detection, forest monitoring, urban monitoring, agricultural monitoring, and natural hazard assessment are discussed, demonstrating enhanced accuracy and scope. Finally, this review identifies persistent challenges such as technical complexities in data integration, issues with data availability and accessibility, validation hurdles, and the need for standardization. It proposes future research directions focusing on advanced AI, novel fusion techniques, improved data infrastructure, integrated “space–air–ground” systems, and interdisciplinary collaboration to realize the full potential of multi-sensor satellite data for robust and timely land surface monitoring. Supported by deep learning, this synergy will improve our ability to monitor land surface conditions more accurately and reliably.

## 1. Introduction

Accurate and timely land monitoring is indispensable for addressing critical environmental, economic, and societal challenges. While this review focuses on satellite-based remote sensing technologies—optical, radar, and LiDAR—due to their unique capabilities for large-scale, repeatable, and synoptic observations, we recognize the complementary role of classical geodetic methods such as a Global Navigation Satellite System (GNSS), precise leveling, and gravimetry. These ground-based techniques provide high-precision point measurements of deformation, elevation, and gravity changes, which are often integrated with satellite data to validate and enhance monitoring accuracy. For instance, GNSS networks are routinely used to calibrate Interferometric SAR (InSAR)-derived ground displacement measurements, while gravimetry contributes to subsurface mass change detection. The synergy between satellite remote sensing and geodetic methods is particularly evident in applications like tectonic motion monitoring and infrastructure stability assessment, where GNSS and leveling data are used to validate the absolute accuracy of satellite observations. However, the scope of this review emphasizes satellite technologies due to their growing dominance in generating spatially continuous, global-scale land monitoring products. Monitoring land use and land cover (LULC) changes is essential for understanding their impact on climate change and plays a crucial role in informing strategies for sustainable development [1]. Furthermore, robust land monitoring supports vital applications such as water resource management [2], natural resource investigation and evaluation [3], urban planning [4], disaster assessment [5], agricultural management [6], biodiversity conservation [7], and economic activity assessment [8], particularly in data-scarce regions [9,10]. Early identification of geological hazards and the detection of potentially hazardous environmental elements for infrastructure like railways also rely heavily on effective land monitoring techniques.

Over recent decades, remote sensing technology has undergone significant evolution, offering unprecedented capabilities for Earth observation. Advancements in sensor technology, satellite platforms, data archives, and processing capabilities, including the integration of artificial intelligence and deep learning, have spurred breakthroughs in remote sensing image processing and analysis techniques such as classification, segmentation, and change detection. The increasing availability of multi-source and multi-modal RS data, encompassing various parts of the electromagnetic spectrum and different resolutions, presents opportunities for more comprehensive monitoring.

**Figure 1:**
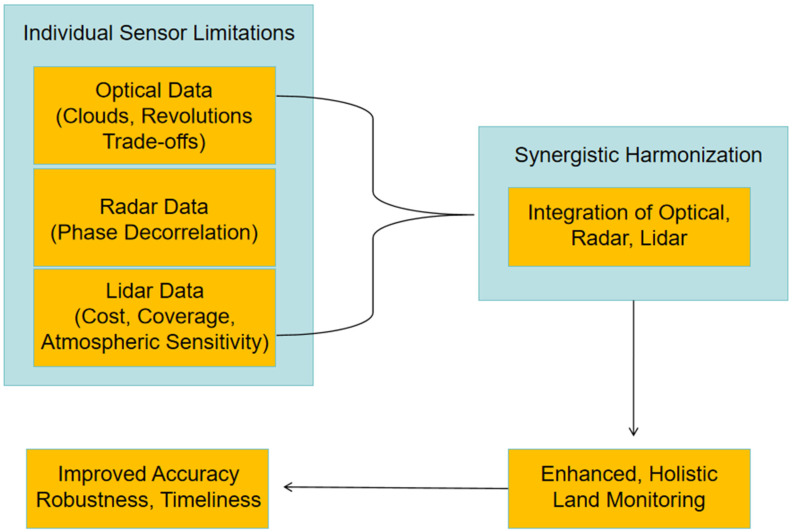
Sketch map of advancing land monitoring through synergistic harmonization of optical, radar, and LiDAR satellite technologies.

Despite these advancements, relying solely on single-source satellite data for land monitoring presents inherent limitations. Individual sensor modalities often face trade-offs between spatial and temporal resolution, or are constrained by environmental factors such as cloud cover (affecting optical sensors) or phase decorrelation due to vegetation (affecting radar). Each modality captures only specific attributes of the land surface, failing to provide a complete characterization of the observed scene. This leads to difficulties in achieving the desired accuracy, timeliness, and robustness required for diverse land monitoring applications. This gap highlights the necessity of approaches that transcend the limitations of single modalities and leverage the complementary strengths of different data types [11,12,13]. Figure 1 shows an illustration of advancing land monitoring through the synergistic harmonization of optical, radar, and LiDAR satellite technologies.

Synergistic harmonization, involving the integration of multi-source remote sensing data, offers a promising solution to overcome these challenges. By combining optical, radar, and LiDAR satellite data, a more holistic and accurate representation of land surface characteristics can be achieved. Optical data provides rich spectral and textural information, which is valuable for land cover classification and characterization [14]. Radar, specifically Synthetic Aperture Radar (SAR), offers all-weather, day-and-night acquisition capabilities and sensitivity to structural and dielectric properties, enabling monitoring through clouds and detecting surface deformation [15]. LiDAR, an active sensor technology, provides precise three-dimensional spatial information and detailed elevation models which are crucial for characterizing vertical structure and terrain [16]. Integrating these modalities allows for the fusion of complementary information, leading to enhanced detection, identification, classification, change detection, and overall improved decision-making performance compared to using individual data sources alone. This synergy enables a more robust and reliable understanding of complex land surface processes and dynamics. Other relevant reviews only discuss the applications of a single technology in a specific area. For example, the review by [16] considers the application of Unmanned Aerial Vehicle (UAV) remote sensing technology in agriculture, forestry, power systems, and the natural environment. Another review by [12] considers the potential application of remote sensing techniques for soil salinity mapping and monitoring.

In contrast, this review aims to provide a comprehensive overview of the advancements in land monitoring achieved through the synergistic harmonization of optical, radar, and LiDAR satellite data. Specifically, the objectives are to review the current techniques and methodologies employed for integrating these distinct data types, analyze their applications across various land monitoring domains, identify the key challenges and limitations associated with multi-sensor data harmonization and processing, and propose future research directions to further advance the field. By synthesizing insights from recent research, this review contributes to the field by offering a structured overview of the state of the art, highlighting best practices, and outlining potential avenues for developing more sophisticated and effective land monitoring solutions.

Due to the abundance of literature in the relevant field, the authors searched and read over a thousand articles worldwide through databases such as Web of Science, IEEE Xplore, Scopus, etc. Following the principles of appropriateness and clarity, the authors selected high-impact-factor literature from the past five years and older publications with significant impact as references for this article.

## 2. Remote Sensing Data and Technologies

Effective land monitoring necessitates the acquisition and analysis of comprehensive spatial data, which are primarily derived from remote sensing technologies operating across various portions of the electromagnetic spectrum [17,18]. The synergistic harmonization of these distinct remote sensing modalities provides a more comprehensive and robust understanding of land surface characteristics and dynamics than relying on a single sensor type. Optical imagery provides rich spectral information, while radar offers all-weather, day-and-night capabilities that are sensitive to structure and dielectric properties. LiDAR, meanwhile, excels in delivering precise three-dimensional structural data. Table 1 summarizes different sensor types and their performances.

Optical, radar, and LiDAR remote sensing systems represent the fundamental pillars of modern Earth observation, each offering unique capabilities that are crucial for understanding the Earth’s surface. These technologies, deployed on spaceborne, airborne, and ground-based platforms, provide multi-source, multi-modal data characterized by varying spatial, temporal, spectral, and radiometric resolutions.

Optical remote sensing captures reflected or emitted electromagnetic radiation in the visible, near-infrared, and shortwave infrared regions [19]. Its strength lies in spectral sensitivity, leveraging the distinct reflectance characteristics of land surface features to differentiate land cover types and assess biophysical properties like vegetation health through spectral indices such as the Normalized Difference Vegetation Index (NDVI) [20,21]. Optical systems range from panchromatic (with high spatial and low spectral resolution) to multispectral and hyperspectral (with lower spatial and higher spectral resolution) [22,23,24,25]. Key platforms include Landsat [26,27,28], Sentinel-2 [5,29,30], Gaofen [31,32,33], and commercial high-resolution satellites like SPOT and Pléiades [34,35]. Preprocessing, including atmospheric correction (e.g., LEDAPS for Landsat) and cloud screening using quality assessment bands, is vital for consistent data quality and multi-temporal analysis [36]. However, optical data acquisition is limited by solar illumination and hindered by cloud cover.

Radar remote sensing, specifically SAR, is an active microwave technology which transmits and receives radio waves. Its distinct advantage is its all-weather and day–night capability, as it remains unaffected by clouds or lighting conditions. Radar backscatter is sensitive to surface physical properties, including roughness, moisture, and dielectric constant [37,38,39]. Different wavelengths (e.g., X, C, L bands) and polarizations, such as those of Polarimetric SAR (PolSAR), provide diverse information [40,41,42]. Sentinel-1, RADARSAT-2, and Gaofen-3 are prominent SAR platforms [43,44]. Preprocessing techniques include speckle filtering and various image processing methods like segmentation, change detection, and classification, often utilizing deep learning approaches [45,46]. InSAR is particularly powerful for monitoring subtle ground deformation, which is critical for geological hazard assessment, although it is susceptible to phase decorrelation in challenging environments [47,48,49].

LiDAR is an active technology that employs laser pulses to measure distances and acquire precise three-dimensional spatial information [50]. Its key strength is the direct measurement of vertical structure, enabling penetration through vegetation canopies to map the ground surface and derive detailed 3D models, digital elevation models (DEMs), and canopy height models (CHMs). LiDAR operates independently of lighting conditions and captures multi-dimensional data, including height, intensity, and waveform information. Spaceborne systems like ICESat-2 and GEDI provide large-scale coverage, while airborne and UAV LiDAR offer higher density and flexibility for regional mapping [51,52]. Preprocessing involves noise filtering, georeferencing, and point cloud classification. Limitations include relatively high cost, typically narrower spatial coverage compared to wide-swath sensors, sensitivity to atmospheric conditions like rain and fog, and limited spectral information.

These three technologies are highly complementary for comprehensive land monitoring due to their distinct sensitivities. Optical data provides rich spectral detail for material identification and vegetation characterization. Radar offers all-weather operational capability and sensitivity to surface structure, moisture, and deformation through InSAR [53]. LiDAR directly measures 3D structure, providing crucial vertical information and high-accuracy terrain models, even under a dense canopy [50]. Combining their strengths can overcome individual limitations, such as using radar or LiDAR in cloudy conditions where optical data is unavailable, or using optical spectral information to enhance radar and LiDAR feature classification [54]. Trade-offs often exist between spatial, temporal, and spectral/structural resolution, requiring careful sensor selection or integration strategies tailored to specific monitoring objectives. The trend towards miniaturization, integration, and intelligence in sensor systems continues to advance their capabilities and accessibility. Preprocessing, while specific to each data type, is universally critical for preparing data for subsequent analysis and fusion, ensuring geometric and radiometric consistency and removing noise or atmospheric effects. Leveraging the synergistic capabilities of optical, radar, and LiDAR data is fundamental for robust and advanced land monitoring applications.

### 2.1. Optical Remote Sensing

Optical remote sensing systems acquire data by measuring electromagnetic radiation reflected or emitted from the Earth’s surface, primarily in the visible, near-infrared, and shortwave infrared portions of the spectrum. The fundamental principle relies on the unique spectral reflectance characteristics of different land surface features. For instance, healthy vegetation exhibits high reflectance in the NIR region due to its cellular structure, while absorbing strongly in the red and blue visible bands to achieve photosynthesis. This differential reflectance across wavelengths forms the basis for distinguishing and analyzing various land cover types and assessing biophysical properties like vegetation health [55,56].

Optical satellite sensors vary in their spatial and spectral resolution, impacting their suitability for different land monitoring tasks. Panchromatic sensors offer high spatial resolution but lack spectral detail, while multispectral sensors provide several discrete spectral bands, allowing for richer feature differentiation at typically lower spatial resolution. Hyperspectral sensors, conversely, capture data across numerous narrow, contiguous spectral bands, enabling detailed spectral analysis and discrimination, but often with the lowest spatial resolution. Common platforms providing optical data for land monitoring include the long-standing Landsat series (TM/ETM+/OLI, the European Space Agency’s Sentinel series (e.g., Sentinel-2), and high-resolution constellations such as SPOT, Pléiades, Pléiades Neo, and WorldView [57,58]. China also contributes significantly with its Gaofen series (e.g., GF-1, GF-2, GF-7) and Fengyun series (e.g., FY-3D with MERSI) [59,60,61]. The fusion of panchromatic, multispectral, and hyperspectral images is often employed to combine the advantages of high spatial and spectral resolution for applications like detailed hazard monitoring [62].

Optical data offers capabilities that are leveraged across numerous land monitoring applications. For land cover classification, multispectral data from Landsat and GF-1 have been used to create global datasets like GLC30 [63]. Hyperspectral data, with its rich spectral information, is particularly valuable, and research focuses on advanced classification techniques such as kernel joint sparse representation, weighted multi-feature approaches, and cluster-based group sparse coding [64]. Dimensionality reduction techniques, like sparse graph learning and group-based tensor models, are crucial preprocessing steps for hyperspectral imagery to handle its high data volume. Vegetation health assessment is effectively performed using NIR bands and spectral indices like the NDVI), which is derived from red and NIR reflectance values. Optical data, including Sentinel-2 and Landsat-8/9, is also used to model the Leaf Area Index (LAI) [65]. Beyond classification and vegetation monitoring, optical data supports diverse applications such as land surface temperature monitoring using thermal infrared bands, mapping hydrothermal alteration minerals using SWIR band ratios (e.g., Sentinel-2 bands 11/12), monitoring surface water resources, wildfire mapping, and severity assessment, and urban flood mapping using high-resolution imagery from Sentinel-2 and PlanetScope [66].

Accurate land monitoring products derived from optical data necessitate rigorous preprocessing, including atmospheric and geometric corrections. Atmospheric correction methods, such as LEDAPS, which is used for Landsat data, remove the effects of atmospheric scattering and absorption, converting raw radiance values to surface reflectance, which is essential for consistent multi-temporal analysis and comparison [67,68]. Cloud screening, often performed using quality assurance bands, is another critical step in the identification and masking of pixels affected by clouds. Geometric corrections ensure accurate spatial positioning and alignment of imagery [69,70].

Despite their versatility, optical remote sensing systems are subject to significant limitations. Their primary limitation is dependence on passive solar illumination, which makes data acquisition challenging during nighttime or in polar regions during winter. Furthermore, optical signals cannot penetrate clouds, which severely restricts data availability in persistently cloudy areas or seasons. The revisit cycle of single optical satellites can also be long, hindering continuous or rapid change monitoring when specific temporal windows are required. While airborne and UAV platforms offer greater flexibility and lower altitude observation, they face limitations related to acquisition costs, flexibility, timeliness, and regulatory constraints. These limitations necessitate the use of multi-source, multi-temporal optical data or integration with other remote sensing technologies to achieve consistent and reliable land monitoring outcomes.

### 2.2. Radar Remote Sensing

Radar remote sensing is an active microwave imaging technology that operates by transmitting electromagnetic radiation and receiving the backscattered energy from the Earth’s surface. A typical radar system comprises a transmitter, an antenna, and a receiver. The antenna broadcasts radio waves, which interact with objects on the ground and are then scattered back to the system, where the receiver decodes the return signal to determine properties such as the range, size, and speed of objects. This backscattering is highly sensitive to the physical and dielectric properties of ground objects, including material composition, humidity, geometry, and surface roughness. Analyzing the changes in these scattering characteristics across multiple temporal acquisitions is fundamental for applications like urban change detection.

Different radar bands, corresponding to various wavelengths, provide distinct information about land surface properties. Commonly used bands include X-band, C-band, and L-band. PolSAR further enhances the information content by detecting the polarimetric scattering characteristics of targets using multiple polarization modes for both transmission and reception. This multi-parameter approach reflects rich microwave polarization scattering information, aiding in the identification and monitoring of specific features and hazards. Satellites such as China’s Gaofen-3, RADARSAT-2, and Sentinel-1 provide diverse radar and polarization features that are widely used in Earth observation. For instance, the Gaofen-3 satellite has demonstrated high accuracy (>85%) for water body extraction under various conditions.

A significant advantage of radar data is its all-weather and day–night imaging capability. Longer radar wavelengths are less affected by atmospheric conditions such as rain, snow, and fog, making radar suitable for continuous monitoring applications. This capability enables reliable observation regardless of cloud cover. Additionally, radar systems are generally considered cheaper to operate and maintain compared to some other remote sensing technologies. These attributes make radar valuable for applications requiring frequent and reliable data acquisition, such as geological mapping and structural analysis, as well as lake ice classification, due to their high microwave reflectivity. Radar sensors can even produce comprehensive 3D models of the Earth’s surface.

Despite its advantages, SAR imagery is inevitably affected by speckle noise, which can reduce image quality and complicate interpretation. While not detailed in the digests regarding the effectiveness of specific filtering techniques, various SAR image processing methods, including segmentation, change detection, and classification using techniques like neural networks and deep learning, are extensively researched to handle the characteristics of SAR data, including noise and the less intuitive visual appearance compared to optical images.

InSAR is a powerful technique for monitoring surface deformation. By analyzing the phase difference between two or more SAR acquisitions of the same area, InSAR can detect subtle ground movements, making it highly relevant for monitoring geological hazards like landslides. Advanced InSAR time-series techniques are employed for detailed deformation analysis. However, InSAR applications face challenges such as phase decorrelation, often caused by dense vegetation or significant ground deformation. To mitigate vegetation-induced decorrelation, using SAR imagery with longer wavelengths (S-band or L-band), shorter temporal baselines, and higher spatial resolution is suggested. For large deformation gradients, alternative or complementary techniques like SAR offset tracking and range split-spectrum interferometry may be employed.

### 2.3. LiDAR Remote Sensing

LiDAR (Light Detection and Ranging) is an active remote sensing technology that utilizes laser pulses to acquire three-dimensional spatial information about targets. The fundamental principle involves measuring the time-of-flight of laser pulses reflected from objects, enabling precise distance determination. This active acquisition method provides unique vertical structure information and high-resolution 3D representations of the land surface and the objects upon it, distinguishing it from passive optical or microwave radar systems.

The inherent characteristics of LiDAR, such as the high monochromaticity, directionality, and coherence of its laser beams, allow for focused energy transmission and high accuracy in data acquisition. A key advantage of LiDAR data is its ability to penetrate vegetation canopies, although gaps in the undergrowth are still needed, enabling the acquisition of ground surface elevation data even in densely vegetated areas. This capability facilitates the generation of accurate digital elevation models (DEMs) and detailed canopy height models (CHMs), which are crucial for applications such as forest inventory, biomass estimation, and hydrological modeling [71,72,73]. Furthermore, LiDAR operation is generally not affected by lighting conditions, providing data acquisition flexibility day and night. It is noted that twilight or nighttime conditions are often preferable for LiDAR measurements. LiDAR data also provides multiple dimensions of information, including height features, laser reflection intensity, and waveform information, which can enhance the accuracy of feature identification and monitoring.

LiDAR systems are deployed across various platforms, including spaceborne, airborne (manned aircraft and UAVs), and ground-based configurations. Spaceborne systems, such as ICESat-1 (GLAS), ICESat-2 (ATLAS), GEDI, and GaoFen-7, offer broad observation ranges and the capability to acquire large-scale 3D information, even in remote regions. These systems demonstrate varying vertical accuracies; for instance, ICESat-1 achieved accuracy at 0.15 m, ICESat-2 achieved accuracy at approximately 0.1 m, and GaoFen-7 reported 0.3 m vertical accuracy, indicating the potential for high-precision products after appropriate preprocessing. Airborne systems, including commercial and research platforms like SLICER, LVIS, MABEL, and various UAV-based sensors (e.g., TerraLuma, LiAir V series, RIEGL VUX-SYS), are widely used for detailed regional-scale mapping and dynamic change monitoring. The accuracy of LiDAR-derived products, such as DEMs and CHMs, is typically high, following standard preprocessing techniques such as noise filtering, georeferencing, and point cloud classification. Applications like above-ground biomass estimation and wetland classification leverage the accuracy of airborne LiDAR data, often in combination with other remote sensing data sources.

Despite its significant advantages, LiDAR data presents certain limitations. One major constraint is the relatively high cost of LiDAR systems and data acquisition compared to passive optical or (some) radar systems. Furthermore, LiDAR systems—particularly airborne and ground-based systems—have limited spatial coverage compared to the wide swath capabilities of many satellite-based optical and radar sensors, though spaceborne LiDAR offers broader coverage, albeit often with discrete footprints or narrow swaths. LiDAR is also highly sensitive to atmospheric interference from conditions such as rain, snow, and fog, which can significantly compromise data quality and effectiveness. Additionally, most widely used LiDAR systems operate in single bands, providing limited spectral information compared to multispectral or hyperspectral optical sensors, which can make direct feature identification challenging and often necessitates integration with optical imagery to enhance monitoring capabilities.

## 3. Synergistic Harmonization Approaches and Techniques

Advancing land monitoring capabilities necessitates the synergistic harmonization of diverse satellite datasets, including optical, radar, and LiDAR modalities. This integration is crucial for leveraging the complementary strengths of each sensor type to derive more comprehensive, accurate, and reliable information about the Earth’s surface [58,74].

**Figure 2:**
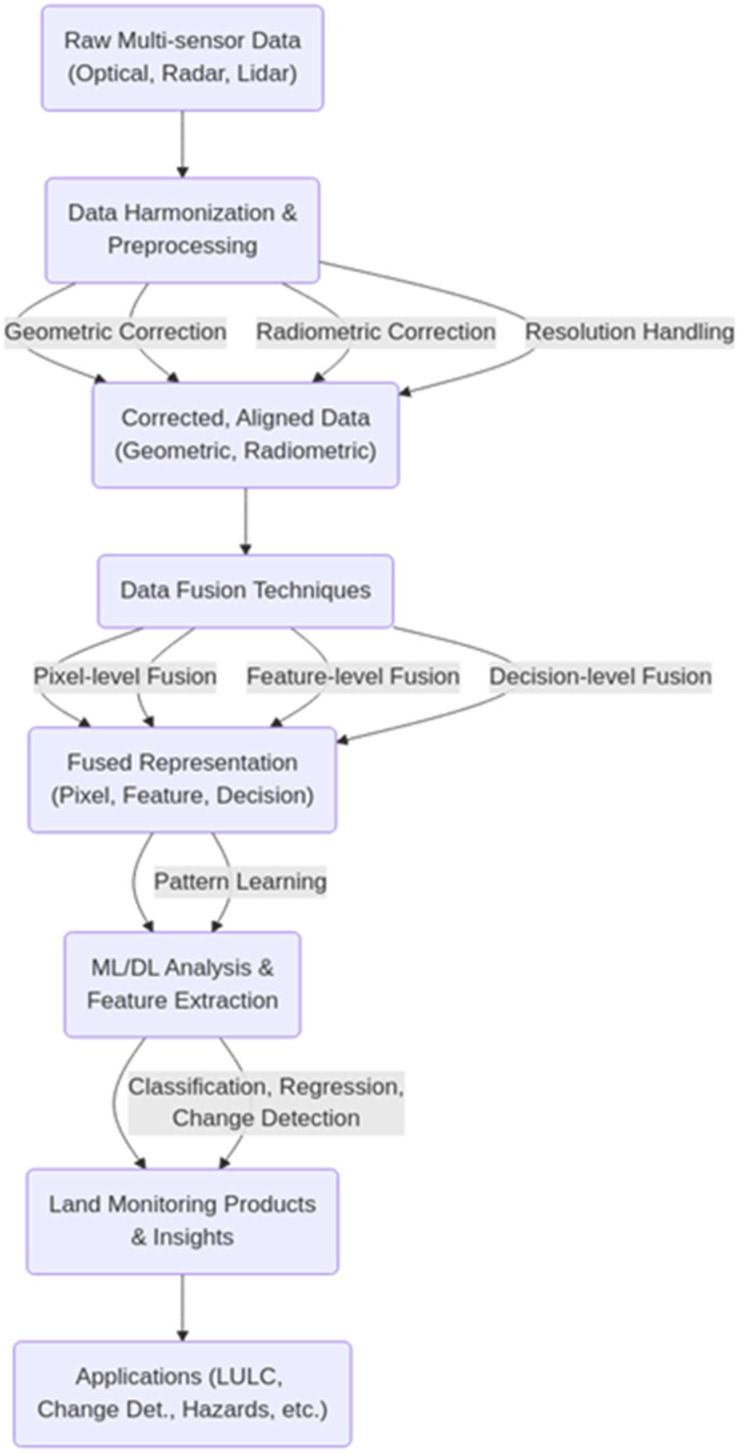
Synergistic harmonization roadmap.

Significant progress has been made in developing methodologies to integrate these diverse data sources. Machine learning, particularly deep learning, has shown great promise and has been successful in multi-modal remote sensing data fusion. Techniques such as multi-attentive hierarchical fusion have improved the accuracy of land cover classification by integrating modality-specific and combined features, as demonstrated by the fusion of hyperspectral and LiDAR data. AI-driven approaches, including those that combine artificial intelligence with human expertise, are essential for improving the accuracy and efficiency of land cover mapping. Figure 2 shows the roadmap of synergistic harmonization.

The process of synergistically combining these heterogeneous data sources involves several key stages, starting with fundamental data harmonization and preprocessing, followed by applying sophisticated data fusion techniques, which are often powered by machine learning and deep learning algorithms.

A critical prerequisite for effective multi-sensor data fusion is rigorous data harmonization and preprocessing. This initial stage addresses the inherent heterogeneity among optical, radar, and LiDAR data, which arises from variations in sensor characteristics, acquisition geometries, atmospheric conditions, and temporal differences. Without proper harmonization, these inconsistencies can introduce artifacts and errors in the fused product, hindering downstream analysis. Key harmonization steps include geometric correction to achieve precise spatial alignment, which is crucial for pixel-level methods, and radiometric correction to normalize signal variations caused by sensor calibration, atmosphere, and terrain. Furthermore, techniques are employed to manage disparate spatial and temporal resolutions, such as hierarchical processing or spatiotemporal fusion models, ensuring data consistency for integrated analysis [75,76]. Table 2 shows different fusion levels and their parameters.

Following harmonization, data fusion techniques are applied to combine the processed data. These techniques are typically categorized based on the processing stage at which fusion occurs: pixel-level, feature-level, and decision-level fusion. Pixel-level fusion directly combines raw data, often aiming to enhance spatial or spectral resolution or integrate different data types like SAR and optical images, though precise alignment is paramount. Feature-level fusion extracts relevant features from individual sources (e.g., spectral indices, texture parameters) and then combines these features, which is particularly useful for handling multi-band data and creating comprehensive input for classification tasks. Decision-level fusion combines the results or decisions derived from processing each source independently, employing methods like voting or evidence theory, which is advantageous for highly heterogeneous data but challenging when resolving conflicting information. The optimal choice of fusion level and technique depends on the specific application and data characteristics, with evaluation based on statistical metrics, reference image comparisons, and source image relationships.

Machine learning (ML) and deep learning (DL) algorithms play a transformative role in synergistic harmonization, moving beyond traditional methods to automatically learn complex patterns and facilitate data integration. Traditional ML algorithms like Random Forest and Support Vector Machines are widely used for classification and analysis, demonstrating effectiveness in tasks like land cover mapping and flood extent mapping by combining diverse inputs. Deep learning, with architectures like Convolutional Neural Networks (CNNs) for spatial feature extraction, Recurrent Neural Networks (RNNs) for temporal data, Generative Adversarial Networks (GANs), and Autoencoders (AEs) for dimensionality reduction and feature learning, excels at handling the high dimensionality and complexity of multi-modal remote sensing data. Recent advancements include Transformer architectures and the development of remote sensing foundation models, leveraging pre-training on diverse modalities to improve generalization and reduce dependence on task-specific labeled data ML/DL can be applied at different fusion levels, from learning fused representations at the pixel or feature level to combining outputs at the decision level, providing flexible frameworks for multi-modal integration [77,78].

Despite the significant progress, challenges persist in synergistic harmonization. Handling the inherent heterogeneity of optical, radar, and LiDAR data, including differences in measurement principles, resolutions, and sensitivities, requires sophisticated preprocessing and potentially specialized model structures. Ensuring data consistency across varying acquisition times and environmental conditions remains difficult. Selecting the most appropriate fusion method or ML/DL architecture is task-dependent and involves trade-offs between accuracy, computational complexity, data requirements (particularly for supervised DL, though transfer learning and human–machine collaboration offer partial solution), and interpretability. The integration of multi-source, multi-scale, and multi-temporal data remains a key research area, requiring robust techniques for spatiotemporal modeling and analysis. The field is actively moving towards AI-driven approaches and cross-modality fusion, highlighting the growing importance of intelligent sensing and interpretation for realizing the full potential of multi-sensor satellite data in land monitoring [79,80].

### 3.1. Data Harmonization and Preprocessing

The synergistic harmonization of diverse satellite data, encompassing optical, radar, and LiDAR modalities, necessitates rigorous data harmonization and preprocessing to mitigate the challenges posed by intrinsic data heterogeneity. This heterogeneity arises from variations in sensor characteristics, atmospheric conditions, illumination, viewing geometry, and acquisition times, which can significantly impact the accuracy of subsequent data fusion and analysis. Effective harmonization workflows are therefore paramount to ensure data consistency and comparability across different sources and acquisition periods.

Preprocessing multi-sensor satellite data involves several critical steps to ensure accurate integration. Geometric correction establishes precise spatial alignment between datasets through registration techniques and geographic coding. This addresses potential misregistration errors that could otherwise distort the final product. For SAR data specifically, this involves correcting geometric distortions such as shadows and overlaps using digital elevation models or machine learning approaches.

Radiometric correction normalizes pixel values across different sensors by accounting for calibration differences, atmospheric effects and terrain-induced illumination variations. Techniques include using surface reflectance products, applying cloud screening and creating median-based annual composites to minimize atmospheric interference. Specialized methods such as GACOS correct atmospheric effects in SAR data, while filters address noise and speckle patterns.

Resolution harmonization handles disparate spatial and temporal characteristics through resampling techniques. The GLOSTFM model’s image pyramid approach is an effective method for the hierarchical processing of multi-resolution data processing [81]. Together, these preprocessing steps form an essential foundation for reliable, synergistic analysis, minimizing artefacts while preserving data integrity for downstream applications such as change detection and pixel-level fusion.

### 3.2. Data Fusion Techniques

Advancing land monitoring through the integration of multi-sensor satellite data necessitates robust data fusion techniques, which are broadly categorized based on the processing stage at which the data are combined: pixel-level, feature-level, and decision-level fusion. Each level presents distinct advantages and is suited for different data types and applications, although they also face unique challenges.

Pixel-level fusion involves the combination of raw data from multiple sources to generate a single output, with the aim of enhancing information content or detecting changes across different acquisitions. This level is often employed to synthesize SAR images with rich texture and structure information, even when fused with optical data, despite the substantial differences in their imaging mechanisms. Techniques at this level include component substitution, modulation-based methods, and multiresolution analysis. A primary goal is often to improve spatial or spectral resolution. For instance, multispectral imagery from different sensors like Landsat, HJ-1, and GF-1 can be combined at the pixel level to enhance land cover classification accuracy [82]. Challenges at the pixel level primarily revolve around precise data alignment and handling scale differences between images.

Feature-level fusion operates by extracting various features from individual data sources and then combining them into unified feature maps for subsequent processing. This approach is particularly beneficial when dealing with a large number of spectral bands, making individual band analysis impractical. Feature extraction methods are often tailored to the characteristics of the specific data sources, which can vary for heterogeneous datasets. Examples of features include coefficient-based transformations like spectral indices (e.g., NDVI), which represent ratios of values across different bands to indicate the reflectance of specific features. These extracted features can then be combined using techniques such as statistical methods or artificial neural networks (ANN). More advanced approaches utilize hierarchical fusion, incorporating attention mechanisms like modality attention to integrate features while highlighting modality-specific characteristics, especially relevant for fusing heterogeneous data like Hyperspectral Imagery and LiDAR. Feature-level fusion is suitable for creating comprehensive feature sets for tasks like classification, as demonstrated by integrating Landsat spectral bands, NDVI, and nighttime light data as covariates in a Random Forest model for land cover change mapping. Challenges include selecting the most informative features and managing potential redundancy among them. Precise pixel-level registration is typically required even for feature extraction.

Decision-level fusion combines the results or decisions obtained from processing individual data sources or applying different algorithms separately. This involves extracting information from each input image independently and then applying decision rules to reconcile differing interpretations and strengthen common findings. Depending on whether the results are expressed as confidence scores (soft fusion) or final decisions (hard fusion), different methods are applied. Common techniques include voting methods, statistical approaches, and methods based on fuzzy logic. An improved Dempster–Shafer (D-S) theory, incorporating weighted synthesis of evidence based on certainty and average support, has been employed for decision-level fusion in applications like urban change detection using multi-temporal SAR imagery, aiming to improve reliability by better handling conflicting information. Decision-level fusion is particularly useful when the input data are very heterogeneous or when independent processing pipelines are already established. A significant challenge lies in effectively handling and resolving conflicting information derived from different sources or algorithms.

The effectiveness of data fusion is assessed through various criteria, including statistical properties of the fused image (e.g., mean, standard deviation, entropy), its relationship with a reference image (e.g., Root Mean Square Error (RMSE), Spectral Angle Mapper (SAM), Peak Signal-to-Noise Ratio (PSNR)), and its relationship with the source images (e.g., Mutual Information (MI), Correlation Coefficient (CC), Relative Dimensionless Global Error in Synthesis (ERGAS), Relative Average Spectral Error (RASE)). A comprehensive evaluation necessitates integrating both subjective and objective assessments, selecting indices based on factors like coverage area, acquisition time, and the specific application [83]. A precise procedure for data fusion techniques can be summarized as follows: problem definition and data preparation, registration, data association, state estimation, model fusion, process refinement, and user refinement.

Data fusion techniques, across all levels, are crucial for applications such as Digital Surface Model/Digital Elevation Model generation, 3D object extraction, land cover mapping, above-ground biomass (AGB) estimation, and environmental change detection. Examples include fusing multi-spectral, Radar, and LiDAR data with machine learning for mangrove ecosystem mapping, combining NAIP and NOAA data for post-disaster damage assessment, and applying spatiotemporal fusion, such as Gauss-Laplace pyramid-based methods, for land surface temperature date. The field continues to evolve with AI-driven techniques and cross-modality fusion receiving significant attention, as highlighted in initiatives like the IEEE GRSS Data Fusion Contests and relevant conference. Object-based analysis can also complement fusion efforts, particularly in high-resolution imagery, by incorporating neighborhood relationships to improve analysis outcomes like damage mapping.

### 3.3. Machine Learning and Deep Learning Approaches

Machine learning and deep learning algorithms have become instrumental in advancing land monitoring by enabling the automatic learning of complex features and relationships from heterogeneous multi-source satellite data. These nonparametric classifiers offer robust alternatives to traditional methods, particularly when dealing with the high dimensionality and large volumes characteristic of modern remote sensing datasets. Their application spans various land monitoring tasks, including land cover mapping, change detection, flood mapping, and wildfire type classification, among others like LAI modeling, canopy height mapping, and mangrove monitoring [84,85].

Traditional ML algorithms, such as Random Forest and Support Vector Machines (SVMs), have been widely applied. RF models, for instance, have been effectively used for continental land cover change mapping, employing a large number of decision trees (e.g., 500) with final classification based on majority vote. RF has also been successfully deployed for urban flood extent mapping using multi-sensor inputs like Sentinel-2 imagery, elevation, and land cover data. When comparing different ML approaches, studies show varied performance; for wildfire type classification, XGBoost demonstrated superior performance compared to Random Forest, achieving higher F1 scores. SVM is also noted as a common ML algorithm used in multi-omics data analysis, highlighting its general applicability in complex data integration tasks.

Deep learning, in particular, excels at learning intricate patterns and hierarchical representations from data. Various DL architectures are utilized depending on the task and data characteristics. CNNs are predominant for spatial feature extraction and pixel-based classification tasks like land cover mapping and semantic segmentation. Specific CNN variants, such as FCNs, have shown higher accuracy in LULC mapping compared to the pixel-based maximum likelihood method. Custom CNN architectures, like MAHiDFNet, which incorporates modality attention and self-attention modules, are designed for enhanced multi-modal fusion and classification. CNNs are also fundamental to DL-based multi-modal data fusion and pan-sharpening. RNNs are suitable for processing sequential data, which is relevant for temporal analysis and change detection. GANs are employed for tasks like change detection and potentially for data generation or super-resolution, including applications in pan-sharpening. AEs are commonly used for dimensionality reduction and learning low-dimensional latent representations, and these properties are applicable in change detection and multi-modal data integration. Deep Belief Networks (DBNs) are also noted for their potential in tasks like high-resolution SAR image classification and change detection. Recent advancements introduce Transformer architectures to remote sensing for tasks like building extraction and pan-sharpening, leveraging their ability to model global dependencies.

The advantages of deep learning include its capacity to handle high-dimensional data and automatically learn complex, hierarchical features, leading to improved accuracy in various tasks like SAR image classification and change detection. However, challenges remain, notably the significant requirement for large, labeled training datasets and the computational cost associated with training complex models. Approaches like human–machine collaboration, where ML models accelerate land cover mapping and manual interpretation assists AI, or using subjective rankings for supervision, help mitigate the reliance on extensive ground-truth data. Model interpretability is another challenge, though techniques like Grad-CAM can visualize feature importance [86].

Transfer learning plays a crucial role in reducing the dependency on large, task-specific datasets by leveraging models pre-trained on vast generic or related datasets. This concept has led to the development of remote sensing foundation models, often based on Transformer or Autoencoder architectures, which are pre-trained on diverse remote sensing modalities. Examples like SatMAE and RingMo demonstrate how pre-training enables these models to generalize effectively to new tasks with less fine-tuning data [87]. Furthermore, the emergence of vision-language models, such as CLIP and GLIP, bridges the gap between image content and textual information, enhancing tasks like classification and object detection by integrating multi-modal understanding, fundamentally addressing the constraints of traditional discriminative models. This shift towards “remote sensing big data + artificial intelligence” represents a key direction for timely and stable monitoring [88].

A precise procedure for machine learning modeling can be summarized as follows: problem formulation and data preparation, model selection and architecture design, regularization and optimization, training and validation, iterative refinement and bootstrapping, followed by evaluation.

The integration of multi-modal data is facilitated by ML/DL through various strategies, including learning separate representations before concatenation, modeling inter-modality relationships, and using model-based or transformation-based fusion approaches. This is reflected in initiatives like contests focused on intelligent interpretation for multi-modal remote sensing application. The continued research into AI technology related to sensor systems underscores the growing importance of ML and DL in processing and analyzing diverse remote sensing data for land monitoring.

## 4. Applications of Synergistic Land Monitoring

**Figure 3:**
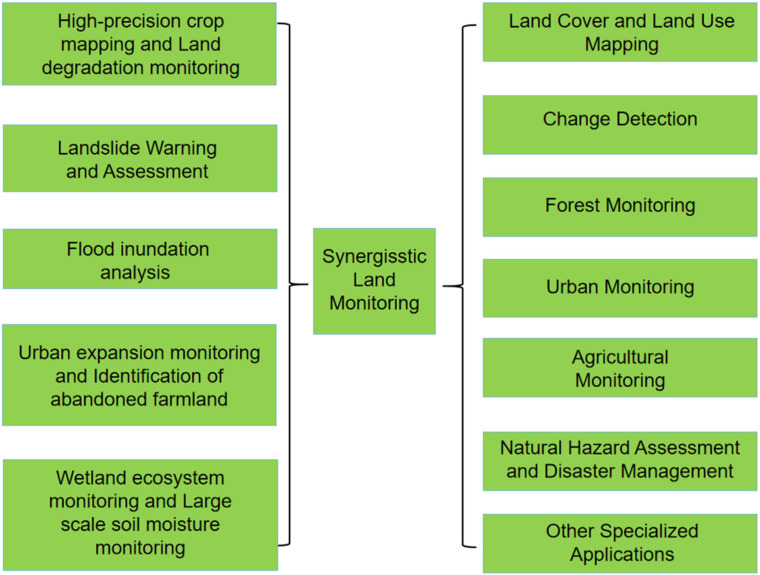
Applications of synergistic land monitoring.

Synergistic harmonization of optical, radar, and LiDAR satellite data represents a transformative approach to land monitoring, offering significant advancements over methodologies relying on single-source data. This integrated strategy leverages the complementary strengths of different sensor modalities—the rich spectral information from optical sensors, the all-weather penetration capabilities and surface property sensitivity of radar, and the precise 3D structural and topographical data that can be obtained using LiDAR. By combining these diverse data types, synergistic approaches enhance the accuracy, detail, and scope of land monitoring products across a wide array of applications [74]. Figure 3 shows the applications of synergistic harmonization in the context of land monitoring.

The benefits of synergistic land monitoring are evident across fundamental applications like land cover and land use (LULC) mapping. While optical data alone can generate LULC maps, integration with structural information from LiDAR and surface properties from radar allows for better differentiation of spectrally similar classes and improved accuracy in complex landscapes. Similarly, in change detection, multi-temporal analysis is fundamental, but the synergy of optical, radar (SAR), and LiDAR data, often combined with advanced AI techniques, provides enhanced robustness and the ability to detect various types of changes, from subtle shifts to significant disturbances like wildfires or urbanization [74].

Despite their power, these approaches face challenges, including data heterogeneity, processing complexity, and the need for advanced algorithms to effectively combine different sensor outputs. Future developments will focus on improving machine learning techniques and integrating ground-based data sources more effectively.

### 4.1. Land Cover and Land Use Mapping

Accurate and detailed land cover and land use (LULC) mapping is fundamental for environmental monitoring, urban planning, and resource management. While LULC maps can be generated from individual sensor data, such as optical imagery used for global products like GLC30 and GLC_FCS30D, or continental mapping efforts like that conducted for Africa using Landsat, these often encounter limitations in distinguishing classes with similar spectral properties or in complex landscapes [63]. Consequently, a primary focus of current research is improving LULC mapping accuracy through the synergistic fusion of data from multiple sensor types.

The combination of spectral (optical), structural (LiDAR), and surface property (radar) information significantly enhances classification capabilities. Optical sensors, particularly hyperspectral (HS) imagery, provide rich spectral signatures that are valuable for identifying materials and land cover types. However, spectral information alone can be insufficient to differentiate targets when spectral variability is high or classes are spectrally similar. This is where data from LiDAR and radar become crucial.

LiDAR systems capture detailed 3D spatial geometry, providing structural information that is complementary to the spectral data from an optical sensor. The joint use of HS and LiDAR data has become a significant area of research, enabling better discrimination of land cover types based on their vertical structure, which is particularly useful for classifying vegetation types, urban areas, and distinguishing impervious surfaces from other bare ground. Studies fusing HSI and LiDAR through advanced techniques like deep learning models have demonstrated effectiveness in distinguishing various land cover classes and improving classification accuracy.

Radar data, such as SAR, provides information about surface roughness, dielectric properties, and structural characteristics, which can penetrate clouds and are sensitive to moisture content and geometric arrangements. The synergy of optical and radar data offers robustness against atmospheric conditions and provides unique insights into surface properties that are not available from optical data alone. For instance, fusing Sentinel-2 optical data with Sentinel-1 radar data has been effectively used for LULC classification, including distinguishing between different forest states like intact, degraded, and regenerating forest [89]. Combining hyperspectral and SAR imagery has also shown improved accuracy in land cover classification through techniques like kernel joint sparse representation and deep learning.

The advantage of synergistic approaches lies in their ability to leverage the strengths of different sensor modalities, overcoming the limitations of single-sensor data. By integrating spectral, structural, and surface property information, synergistic fusion enhances the discriminability of complex land cover classes, improves accuracy in challenging landscapes, and allows for more detailed and reliable LULC mapping. This is particularly beneficial for mapping classes that exhibit significant within-class variability in one modality but homogeneity in another, or for applications requiring differentiation of fine-grained LULC categories, such as those detailed in the GLC_FCS30D dataset with its 35 subcategories. The development of advanced machine learning and deep learning techniques is crucial for effectively processing and fusing these multi-modal datasets, further contributing to the improved accuracy and detail of LULC maps derived from synergistic data. The effectiveness of these synergistic methods has been demonstrated across various geographic locations and contexts [90,91].

### 4.2. Change Detection

Land cover change detection is a fundamental application in remote sensing, which is crucial for monitoring dynamic Earth processes and informing land management decisions. This involves comparing images of the same area acquired at different times to identify areas that have undergone transformation. Various types of land cover change can be detected and monitored, including deforestation, urbanization, disturbance events such as wildfires, and even subtle shifts in infrastructure like roofing and road networks. Beyond physical land cover, change detection techniques are also applied to track indicators of regional development and changes in biodiversity by integrating remote sensing data with ecological information.

Historically, change detection has relied on traditional methods such as Change Vector Analysis, sometimes applied in spaces derived from posterior probabilities [92]. Continuous change detection algorithms have also been employed to distinguish between areas of persistent stability and those undergoing change over time. With advancements in computing and data availability, artificial intelligence (AI)-based change detection techniques have emerged, significantly enhancing the automation and intelligence of these processes across numerous applications. Specific AI models and paradigms tailored for change detection include “Time-Travelling Pixels” and a new learning paradigm designed for foundation model-based remote sensing change detection. AI methods facilitate generating different types of change detection maps, such as binary maps indicating change or no change, one-class maps highlighting the appearance or disappearance of specific objects, from–to maps detailing the conversion between classes, and instance maps delineating the boundaries of each change event. The integration of AI techniques allows one to handle complex queries, including change captioning, category-specific quantification, and precise change localization.

The use of multi-temporal data from different sensors is pivotal for improving change detection accuracy and robustness. By analyzing satellite imagery acquired over extended periods, researchers can track changes occurring over various timescales, from yearly shifts in regional development and continuous monitoring to changes over 15-year, 20-year, or even 50-year intervals. Long time-series of satellite Earth observation data are particularly valuable for detecting and assessing processes like forest disturbance and recovery. Synergistic approaches, combining data from multiple sensor modalities such as multi-spectral, radar (SAR), and LiDAR approaches, alongside machine learning methods, have been demonstrated for monitoring specific changes like mangrove extent and canopy density. For instance, fusing features extracted from multi-temporal SAR images using methods like the improved Dempster–Shafer algorithm has been applied effectively for urban change detection [93].

Despite the advantages of multi-temporal and multi-sensor data synergy, challenges remain. Handling heterogeneous data from different sources requires sophisticated techniques, and achieving reliable spatiotemporal fusion to generate dense time-series products with fine spatial resolution, although beneficial for change detection, presents significant hurdles that hinder practical applications. Improving unsupervised change detection methods, particularly for data like SAR imagery, is an ongoing area of research. Efforts are also directed towards high-resolution change detection, even when only low-resolution labels are available. The increasing volume and complexity of multi-temporal, multi-sensor data necessitate the development of advanced algorithms, including parallel processing implementations, to efficiently handle change detection tasks. Specialized tools and datasets, such as those developed within contexts focused on change detection in high-resolution and multi-temporal optical image, and generative models for creating annotated time-series datasets, continue to drive progress in this field.

### 4.3. Forest Monitoring

Remote sensing plays a critical role in modern forest monitoring systems, contributing to initiatives such as providing comprehensive tools for global land cover analysis and change detection. Integrating remote sensing with ecological and evolutionary studies further enhances the capacity to monitor forest ecosystems by capturing detailed structural, compositional, and functional attributes. Earth observation analytics platforms offer detailed products including forest extent, type, Leaf Area Index, Tree Canopy Density, vegetation canopy height, above-ground biomass, and assessments of canopy disturbance and recovery [50,94,95]. Recent datasets reveal significant changes in forest cover globally, with one study indicating a decline of approximately 2.5 million square kilometers over 37 years. Similarly, studies focused on high-biomass regions, often associated with forests, have observed declining trends, showing good agreement compared with established forest maps.

Synergistic harmonization of data from optical, radar, and LiDAR satellites offers substantial improvements in forest monitoring accuracy, reduces uncertainties in biomass estimation, and enhances disturbance detection. LiDAR data provides accurate measurements of a forest’s three-dimensional (3D) structure, delivering crucial information on parameters like vegetation canopy height, which is fundamental for estimating above-ground biomass. Optical sensors, utilizing various spectral bands such as infrared and ultraviolet, provide insights into forest health by measuring chlorophyll content, and are effective for tracking disturbances like forest fires and mapping the extent of forest cover [96]. Radar data complements these findings by providing information sensitive to forests’ structure and moisture. Radar is capable of penetrating clouds and providing consistent monitoring, which is also valuable for biomass estimation and tracking the structural changes associated with disturbances.

The integration of these modalities allows for a more comprehensive analysis. For example, assessing canopy disturbance and recovery is improved by combining the structural sensitivity of LiDAR/radar with the spectral information from optical sensors. Dedicated research on forest disturbance monitoring focuses on the temporal and spatial detection of disturbances, identification of causative factors, and analysis of their impact. Multi-modal, multi-scale benchmarks, such as FoMo-Bench, are being developed to support foundation models for forest monitoring tasks, including classification, segmentation, and detection, highlighting the increasing reliance on integrated datasets.

Despite the advancements provided by synergistic data, challenges persist with regard to forest monitoring. Accurately separating specific forest types can be challenging due to variations within types and similarities between different categories across sensor modalities. Detecting subtle changes, such as early signs of disease, minor defoliation, or gradual structural degradation, requires high sensitivity and frequent observations from complementary sensors. While synergistic data provides richer information (e.g., combining spectral properties from optical with structural details from LiDAR/radar), these complexities necessitate advanced analytical techniques and continued research to fully exploit the potential of integrated satellite observations for precise and detailed forest characterization and change monitoring.

### 4.4. Urban Monitoring

Effective urban monitoring is paramount for sustainable development, planning, and environmental management. Synergistic harmonization of diverse satellite remote sensing data significantly enhances the accuracy and scope of urban area mapping and analysis. By integrating information from optical, radar, and LiDAR sensors, improved urban mapping accuracy is achieved, which is essential for applications such as change detection in dynamic urban landscapes. Decision-level fusion methods, which leverage multisource and multiscale Earth observation data, have demonstrated superior performance in urban area mapping compared to using single data sources.

The combination of spectral, textural, and elevation information provided by synergistic data sources enables a more precise delineation of urban features and facilitates detailed 3D city modeling. Optical data provides spectral characteristics which are valuable for identifying different urban surfaces, while radar data offers insights into structure and texture; such insights are useful for measuring building height and width and assessing infrastructure conditions. LiDAR, in particular, yields highly accurate elevation data, which is crucial for generating detailed 3D representations of urban landscapes, allowing for precise visualization of building placement and road systems. This integrated approach is vital for monitoring urban growth in complex environments, where traditional methods based on single data types may struggle with spectral confusion or structural complexity [97].

Beyond basic mapping, harmonized data supports the monitoring of various urban dynamics. Tracking the increase in impervious surfaces, a key indicator of urban expansion, can be effectively achieved using time-series analysis of remote sensing data. Studies show significant relative increases in impervious surfaces over time, highlighting the rapid pace of urban growth. Multi-temporal SAR image fusion, for instance, can be applied for urban change detection, identifying alterations in the urban landscape. Annual assessment of urban land cover growth is possible at national and regional scales using comprehensive land cover-monitoring products, including the identification of landscape visual changes such as the conversion of natural or agricultural lands into built-up areas [98].

Synergistic monitoring is also crucial for addressing specific urban environmental challenges. High-resolution Land Surface Temperature (LST) data, potentially derived or refined through fusion models like GLOSTFM, is indispensable for monitoring urban heat islands, informing urban planning and mitigation strategies. Research confirms a positive correlation between urban development indicators like the Normalized Difference Built-up Index (NDBI) and LST, illustrating the impact of urbanization on increasing surface temperatures. Furthermore, machine learning models applied to satellite imagery enable the mapping of urban flood distribution, assisting decision-makers in resource allocation for prevention, resilience, and recovery efforts. Remote sensing is also employed globally to track critical environmental parameters like air pollution and CO_2_ emissions across numerous cities.

The complexity of urban environments poses significant challenges for mapping, particularly in dense areas. These challenges include differentiating between materials with similar spectral signatures, accounting for complex building geometries and shadow effects, and capturing rapid vertical and horizontal changes. Integrating diverse data sources helps mitigate these issues by providing complementary information. Moreover, the fusion of remote sensing data with ancillary data sources, such as Points of Interest (POI) data and street view images, offers new opportunities for detailed urban analysis, including the classification of urban functional areas by linking spectral and structural information to human activities and facilities. POI attributes are closely tied to functional facilities, while street view images provide a ground-level, human-centric perspective, offering diversified and complementary environmental descriptions. Addressing the challenges of mapping dense urban areas requires sophisticated fusion techniques that can handle heterogeneity and integrate multi-modal data effectively [99].

### 4.5. Agricultural Monitoring

Remote sensing plays a crucial role in modern precision agriculture by providing spatially varied data with big data characteristics that are essential for operational management. Synergistic data fusion, integrating information from optical, radar, and LiDAR satellite sensors, significantly enhances agricultural monitoring capabilities, leading to more accurate crop classification, improved soil moisture estimation, and better yield prediction. This integrated approach leverages the unique strengths of each sensor type: optical data primarily provides information about vegetation health and phenology; radar provides insights into moisture content and surface properties; and LiDAR contributes detailed structural and topographical information.

Optical sensors are widely used to assess plant health and growth status. Healthy vegetation exhibits high reflectance in the near-infrared spectrum, a property utilized by vegetation indices such as NDVI and the Enhanced Vegetation Index (EVI). These indices, along with others like leaf chlorophyll content retrieval from multispectral data, are key to quantitative monitoring of crop growth and assessing overall plant vitality. Optical data is fundamental for tracking crop phenology within an agricultural monitoring program [100].

Radar systems offer distinct advantages, particularly their ability to penetrate clouds and operate irrespective of daylight, providing consistent monitoring capabilities. Radar is highly effective for mapping soil moisture, which is critical for irrigation management and assessing agricultural drought. Beyond moisture, radar data is valuable for monitoring crop growth stages, detecting disease and pest outbreaks, and contributes to evaluating potential yields. Specific applications include the assessment of diseases like cotton Verticillium wilt.

LiDAR technology provides precise three-dimensional structural data about crops and the underlying terrain. This includes accurate topographical data vital for optimized watershed and field management, as well as measurements of crop height, density, and canopy structure. This structural information is crucial for understanding crop development and biomass, which are key inputs for yield estimation and management.

The synergy of these data types delivers enhanced performance across key agricultural monitoring tasks. For crop classification, combining spectral information from optical sensors, structural details from LiDAR, and dielectric/moisture information from radar allows for more robust and accurate differentiation of crop types and land cover, including specific crops like maize and soybean mapped at high resolutions. Tools like the Global Dynamic Land Cover product are applicable for overall crop monitoring. Improved soil moisture estimation benefits significantly from radar’s direct sensitivity to moisture, complemented by optical data to account for vegetation cover effects and LiDAR data for topographical influences on water distribution. Better yield prediction is achieved by integrating multi-temporal data capturing phenological stages (optical), structural changes (LiDAR), and stress indicators like moisture status (radar) and vegetation health indices (optical) throughout the growing season. Furthermore, geospatial prescription maps derived from integrated remotely sensed data enable precise aerial application of materials, supporting site-specific treatment and increasing efficiency in precision agriculture. The increasing expanse of croplands globally underscores the importance of these advanced monitoring techniques.

### 4.6. Natural Hazard Assessment and Disaster Management

Satellite remote sensing plays a crucial role in natural hazard assessment, disaster monitoring, and facilitating timely response and recovery efforts. Synergistic fusion of data from multiple sensors, including optical, radar, and LiDAR, provides more comprehensive and accurate information for these critical applications [101].

Radar, particularly SAR, is widely employed for mapping the extent of flooding, offering advantages like all-weather capability and sensitivity to surface water. Applications extend to urban environments, where satellite images, often processed with machine learning techniques, are utilized to create urban flood distribution maps, supporting planning, mitigation, and post-disaster recovery. SAR imagery is also valuable for monitoring other disaster types, such as detecting the center of partially covered tropical cyclones [101].

Interferometric SAR (InSAR) is particularly effective for detecting and monitoring ground deformation associated with geological hazards. Satellite radar remote sensing is applied to landslide detection and monitoring, a phenomenon inherently linked to subtle ground movements. Furthermore, InSAR, when integrated with optical and LiDAR data, enables the early identification of serious geological hazards and quantitative monitoring of their movement. This integration allows for a qualitative assessment of potential geohazards from optical and LiDAR data combined with the precise deformation measurements from InSAR. Specific studies have demonstrated the utility of remote sensing for landslide extraction.

Optical and LiDAR data are fundamental for damage assessment following natural disasters due to their ability to provide high-resolution visual and structural information. Post-disaster building damage assessment is a common application of remote sensing images. The fusion of different types of imagery is gaining increasing recognition for improving the accuracy of such assessment. Recent advancements, such as new AI models, are being developed to expedite damage assessments and recovery efforts, notably for events like tornadoes. Remote sensing also facilitates the assessment of broader societal impacts, such as changes in nighttime lighting in the aftermath of earthquakes, using data from satellites like SDGSAT-1 [102].

Beyond floods and geological hazards, remote sensing is vital for monitoring wildfires. This includes classifying wildfire types, which supports real-time monitoring and response. Services like the Rapid Burned Area and Severity Mapping (R-BAM) provide crucial information about wildfire’s extent and impact. Near-real-time active fire data from sensors like VIIRS, distributed by systems such as NASA’s FIRMS, are essential for monitoring ongoing fire activity [103].

While remote sensing data provides invaluable information for hazard assessment and disaster management, effectively integrating this hazard-specific data with other critical information sources, such as dynamic weather data or detailed terrain models, presents inherent complexities. Although the provided sources highlight the benefits and methods of fusing different remote sensing modalities (optical, SAR, LiDAR) for enhanced hazard analysis, they do not specifically detail the challenges associated with integrating hazard output data with external, continuously updating datasets like weather forecasts or disparate terrain elevation products. Such integration is often necessary for comprehensive risk modeling and operational response, but requires addressing issues related to data heterogeneity, timeliness, format compatibility, and georeferencing accuracy across diverse sources.

### 4.7. Other Applications

Beyond core land cover and change mapping, the synergistic use of remote sensing data extends to numerous other specific environmental and infrastructure monitoring domains. In coastal management, for instance, satellite-based Earth observation analytics are vital for detailed assessment and monitoring activities. Specific applications include monitoring the extent and changes of mangrove forests, inventorying aquaculture sites, and assessing the health and status of coastal habitats.

Another critical area benefiting from remote sensing is railway hazard monitoring. While traditional methods, such as integrated video surveillance systems, are deployed in key control areas like mountainous terrain or tunnel–bridge junctions, they suffer from limitations including coverage gaps and visual obstructions from buildings or vegetation. These systems, often relying on red and laser integrated high-definition video, provide valuable ground-based data but cannot fully cover the required monitoring zone, which is typically 500 m on both sides of the railway line, due to cost and line-of-sight constraints. Satellite-based remote sensing offers a complementary solution by providing broader, systematic coverage. The synergy of optical data (for visual assessment of land use, vegetation encroachment), radar data (specifically InSAR for detecting subtle ground deformation indicative of landslides or subsidence), and LiDAR data (for precise 3D mapping of terrain, infrastructure, and vegetation clearance) can significantly enhance the early identification and continuous monitoring of geological and environmental hazards impacting railway safety across extensive networks, overcoming the spatial limitations of ground-based systems [104].

Remote sensing technologies are also applied in a range of other fields, such as energy corridor monitoring and assessing habitat disturbance and reclamation in the energy sector. They contribute to groundwater depletion assessment, the delineation of intermittent rivers and ephemeral streams, and the evaluation of road network characteristics. Advanced applications even include leveraging visual language models trained with geospatial data for tasks like mapping administrative boundaries and geocoding. These varied examples underscore the broad applicability of remote sensing, with the integration of multi-source satellite data offering enhanced capabilities for monitoring complex systems and supporting decision-making across numerous domains [105].

## 5. Challenges and Future Directions

Advancing land monitoring through the synergistic harmonization of optical, radar, and LiDAR satellite data presents a complex landscape characterized by significant challenges and promising future opportunities [54,74,106].

Multi-sensor land monitoring presents significant technical challenges due to the inherent diversity of optical, radar and LiDAR data. Integrating these disparate data types, each with unique spectral, spatial and temporal characteristics, noise profiles, and geometric properties, requires sophisticated processing techniques. Key difficulties include the precise co-registration of high-resolution datasets, the pan-sharpening of very-high-resolution optical imagery, and the resolution of various preprocessing issues, such as the reduction of SAR speckle noise, the decorrelation of the InSAR phase, and the effects of urban shadows. These technical complexities are exacerbated by the growing demand for high-frequency monitoring with improved spatial and temporal resolution, which is driving the need for more efficient and robust fusion algorithms. Current limitations include handling non-registered data streams, managing the computational demands of global-scale processing (even with platforms such as Google Earth Engine) and developing algorithms capable of generalizing across diverse geographic locations and sensor modalities. Additional persistent challenges include optimizing data representations for multi-modal integration and developing effective methods for imputing missing data. Table 3 shows the challenges of synergistic harmonization.

The challenges of synergistic harmonization can hinder the precise positioning of research fields, leading to research deviating from core objectives or resource misallocation. Spatial mismatch can be resolved by matching problem scale and governance scope through synergistic harmonizing.

Data availability and accessibility, while improving with the advent of free datasets like Landsat and Sentinel-2 and platforms such as GE, still present significant limitations, especially for advanced AI-based synergistic analysis. A scarcity of large, open SAR datasets suitable for robust AI training is noted. Existing datasets often lack the diversity of change types needed for comprehensive analysis in geographically varied areas. The creation and maintenance of high-quality, large-scale datasets remain challenging, and the usability of some pre-training datasets varies. Limitations in real-time data availability also constrain operational applications. Overcoming these limitations requires promoting open data policies, particularly for less common or commercially held datasets, and improving data infrastructure for efficient storage, management, distribution, and processing.

Robust validation and accuracy assessment are crucial but complicated by the diversity of tasks and datasets. A key limitation is the scarcity of high-quality ground observation data across diverse environments, hindering comprehensive validation and model generalization. The reliance on ground-truth data in current deep learning models is a specific challenge. Progress is being made through establishing standards and technical systems for quantitative remote sensing product validation and employing various quantitative metric. Alternative validation sources, such as citizen science data and platforms like Geo-WIKI leveraging community input, offer promising avenues to supplement sparse ground truth data and enhance validation across varying conditions. Incorporating human knowledge is also vital [107,108,109,110,111].

Despite efforts in specific applications like continental-scale land cover change mapping using standardized approaches within GEE, the field of synergistic land monitoring, especially in rapidly advancing areas like remote sensing foundation models, still lacks fully standardized methods. Establishing robust guidelines, protocols, and benchmarks is critical for ensuring consistency, reliability, and comparability across diverse studies and applications involving multi-sensor data fusion, processing, and accuracy assessment. A concerted effort is needed to develop comprehensive standards that address the unique challenges presented by fusing optical, radar, and LiDAR data. Table 4 shows the Advancements or opportunities of synergistic harmonization.

The field of synergistic land monitoring is undergoing rapid transformation thanks to several key developments. Artificial intelligence, particularly deep learning architectures such as vision-language models, is transforming how we analyze remote sensing data for applications ranging from disaster response to land cover classification. Meanwhile, data fusion techniques are advancing, allowing them to handle increasingly complex datasets, with progress being made in managing fuzzy data, providing reliability estimates, and developing unified deep learning frameworks for multi-modal integration.

Technological advancements are driving this evolution, including the miniaturization of sensor systems and their enhanced capabilities, as well as next-generation satellite constellations such as CO3D and Pléiades Neo Next. The growing volume of data is being addressed through cloud computing and big data analytics, while integration is expanding beyond satellites to incorporate UAVs, ground sensors, and GNSS data.

Future research priorities will focus on creating comprehensive “space–air–ground” monitoring systems through the AI-powered integration of multi-source data. Key application areas include precision agriculture using dual-function UAVs, improved geological hazard detection through multi-technology integration, and global urban environmental analysis. Methodological advances emphasize spatiotemporal pattern recognition, web-based visualization systems and optimized machine learning for high-level data fusion.

These developments are fostering new interdisciplinary collaborations, particularly at the intersection of remote sensing and ecology/climate science, to address complex environmental challenges. The convergence of technological, methodological and application-focused innovations is creating more robust frameworks for global land monitoring, which will ultimately support sustainable resource management and evidence-based policymaking [112,113,114,115].

### 5.1. Technical Challenges

Advancing land monitoring through the synergistic harmonization of optical, radar, and LiDAR satellite data faces significant technical challenges. These challenges primarily arise from the complexities of integrating diverse data types, the substantial computational demands imposed by large multi-source datasets, and the imperative to develop more efficient, robust, and interpretable fusion and analysis algorithms. The inherent diversity and heterogeneity across different sensor modalities pose fundamental challenges for data integration. Remotely sensed data from optical, SAR, and LiDAR sensors exhibit distinct spectral, spatial, and temporal characteristics, as well as varying levels of noise, geometric distortions, and anti-interference capabilities. Integrating very-high-resolution PAN and multispectral images, for example, requires addressing the discontinuous and heterogeneous spectral and spatial features, as one pixel in the MS image may correspond to multiple pixels with variant spectral features in the PAN band—necessitating precise registration and advanced pan-sharpening techniques. Furthermore, integrating new data sources such as UAV or social media data adds additional layers of complexity. Individual sensor technologies also present inherent limitations, such as those associated with InSAR, which must be considered when relying on these data sources [116,117].

Beyond the complexities of integration, significant preprocessing hurdles exist. SAR imagery is particularly affected by speckle noise and geometric distortions like shadows and layovers. Atmospheric effects can also introduce noise and artifacts, especially for InSAR, where phase decorrelation caused by vegetation and residual topographic phase in areas with high buildings or steep slopes can result in phase unwrapping errors. Environmental complexities—such as building shadows in urban areas with complex hydrology, or the challenge of normalizing local contexts in images—further complicate data processing and analysis. Ensuring the accuracy and consistency of derived products, such as land cover classifications across diverse landscapes, while accounting for data acquisition difficulties in some regions, presents another substantial challenge. Moreover, meeting high-frequency inspection needs for certain applications requires data with high spatial–temporal resolution, which is often difficult to acquire directly [15,118].

The global scale of multi-source remote sensing data results in substantial computational demands for processing and analysis. The computational cost of processing large datasets is considerable, and achieving efficiency in techniques like pan-sharpening remains an area for improvement. Utilizing large-scale processing platforms, such as Google Earth Engine (GEE), introduces challenges related to the necessity of programming skills and the limitations of built-in functions [119].

Addressing these challenges necessitates the development of more efficient, robust, and interpretable fusion and analysis algorithms. This includes designing advanced fusion algorithms capable of effectively handling the complex relationships within diverse data streams. Developing algorithms that can generalize and perform effectively across different kinds of image data from various locations is a key barrier to widespread adoption. Furthermore, finding optimal data representations for the diverse characteristics of remote sensing data remains an ongoing challenge—especially as efforts expand to integrate multiple modalities, including vision, language, and audio, using varied model architectures. Improving the interpretability of these complex algorithms is also crucial for building trust and facilitating scientific understanding [120].

### 5.2. Data Availability and Accessibility

Effective synergistic land monitoring is fundamentally contingent upon the availability and accessibility of diverse remote sensing data. Significant progress has been made with the increasing availability of freely accessible data sources, such as Landsat and Sentinel-2 imagery, which can be readily accessed and processed through platforms like Google Earth Engine. Furthermore, specific global land cover datasets, including GLC_FCS30D and others, are made accessible through various repositories such as GEE, Zenodo Open Science Data repository, CASEarth thematic data system, Geo-WIKI, and dedicated websites requiring registration. Technological advancements are also contributing to the overall increase in data availability, providing expanded opportunities for analysis and exploration. International cooperation is also fostering data sharing, exemplified by initiatives like the MOU for sharing China’s natural resources land satellite data for scientific research. Commercial providers supplement public archives by offering extensive imagery collections and guaranteed priority access for specific needs [60].

Despite these positive developments, considerable challenges regarding data availability and accessibility persist, particularly for advanced applications like AI-based synergistic analysis. There remains a limited availability of truly open datasets, and specifically, there is a scarcity of large SAR datasets suitable for training robust artificial intelligence models. Existing datasets, particularly for tasks such as change detection, often contain a restricted number of change types, rendering them insufficient for comprehensive analysis in geographically diverse areas with complex land cover dynamics. The creation and maintenance of large, high-quality datasets, which are essential for developing sophisticated monitoring techniques, continue to present significant challenges. Moreover, the accessibility and usability of many existing pre-training datasets can vary significantly, posing practical hurdles for researchers. Foundational data limitations also include instances where data is not available in real time, which can be a critical constraint for time-sensitive monitoring applications [121].

Addressing these challenges necessitates a stronger emphasis on open data policies to facilitate broader access to diverse remote sensing data streams, especially for less common or commercially held datasets. Concurrently, there is a critical need for improved data infrastructure that can support the storage, management, distribution, and processing of vast volumes of multi-sensor satellite data more efficiently. Overcoming these limitations is crucial for supporting wider research and application in synergistic land monitoring, enabling the development of more accurate, comprehensive, and timely land cover change detection and monitoring capabilities [122].

### 5.3. Validation and Accuracy Assessment

Ensuring the reliability and accuracy of land monitoring products derived from the synergistic use of optical, radar, and LiDAR satellite data presents significant validation challenges. The inherent diversity of tasks and datasets involved complicates the process of accuracy assessment, despite ongoing efforts to standardize evaluation benchmarks. Furthermore, limitations in the availability of high-quality ground observation data across diverse environmental conditions pose difficulties for comprehensive validation and can constrain the generalization ability of developed models. The complexity and importance of rigorous accuracy assessment are highlighted by various land cover mapping initiatives [57].

To address these challenges, the field emphasizes the need for rigorous accuracy assessment methods and the development of standardized validation systems. The creation of “authenticity test standards and technical system for quantitative remote sensing products” and “technology system for product validation and algorithm test of GF common products” signifies a move towards establishing robust frameworks for product verification [123].

Specific quantitative metrics are routinely employed to evaluate product accuracy. These include, but are not limited to, overall accuracy, Kappa coefficient, confusion matrices, user accuracy, producer accuracy, and Area-Under-the-Curve statistics. Reported accuracies for land cover products demonstrate a range influenced by factors such as classification method and class aggregation level. For example, LULC maps derived using ML methods achieved overall accuracies exceeding 80.74% in 2001 and 90.76% in 2021, while an FCN approach yielded accuracies over 93% in 2001 and 98% in 202. The GLC30 dataset reported overall accuracies increasing from 80.33% (Kappa 0.76) in 2000 to 85.72% (Kappa 0.82) in 2020, while another global product achieved 80% accuracy at class level 1 on each continent. A European initiative reported validation accuracy of 86% for 13 land cover classes, rising to 89% when aggregated to 10 classes. Rigorous validation often involves quantifying accuracy with large sample sets, such as the over 84,000 validation samples used for the GLC_FCS30D dataset, which also demonstrated consistency through comparison with other datasets. Furthermore, comparing results with independent external data sources, such as FEMA flood zones for flood extent maps, provides additional validation.

Beyond traditional methods, alternative validation sources offer potential benefits. Citizen science data, collected by non-experts, can supplement sparse ground truth data, particularly in remote or inaccessible areas. Platforms designed for inter-comparison and validation, such as Geo-WIKI, leverage community input and citizen science approaches to enhance the validation process for land cover product. Such approaches can contribute to a more comprehensive and diverse validation dataset, improving the understanding of product performance across varying conditions.

### 5.4. Standardization and Best Practices

Advancing land monitoring through the synergistic harmonization of optical, radar, and LiDAR satellite data necessitates the establishment of robust guidelines, protocols, and standardized benchmarks. The development of such standards is critical to ensure consistency, reliability, and comparability across diverse studies and applications involving multi-sensor data fusion, processing, and accuracy assessment. While the field, particularly in rapidly evolving areas like remote sensing foundation models, still exhibits a lack of fully standardized methods, the importance of establishing such practices is widely acknowledged in the existing literature, including relevant review papers. Efforts towards standardization are evident in specific applications, such as the demonstration of standardized approaches for continental-scale land cover change mapping utilizing Landsat data within the Google Earth Engine platform. This exemplifies how implementing consistent methodologies can enhance the scalability and reproducibility of land monitoring efforts. However, to fully leverage the potential of synergistically combined optical, radar, and LiDAR data, a concerted effort is required to develop and adopt comprehensive standards that address the unique challenges and opportunities presented by the fusion of these distinct data modalities. Such standardization is paramount for fostering scientific rigor and enabling the wider adoption and application of multi-sensor land monitoring techniques [54,74,124].

### 5.5. Emerging Trends and Future Research Opportunities

Technological innovation and interdisciplinary integration are bringing about transformative changes in synergistic land monitoring. The field is witnessing three major developments that are reshaping its landscape [113].

Firstly, artificial intelligence is transforming data analysis with new architectures such as vision-language models and generative AI, enabling significant advances in rapid damage assessment and land cover classification. These AI systems are becoming increasingly adept at handling complex, multi-source datasets with greater accuracy.

Secondly, advanced fusion techniques are emerging to overcome the current limitations. Next-generation methods now incorporate fuzzy data handling and reliability estimation, while hardware-level solutions such as multi-mode fusion imaging systems simplify processing. Researchers are developing unified deep learning frameworks to seamlessly integrate the growing number of sensor modalities.

Thirdly, infrastructure advancements are supporting these technical developments. Cloud-native platforms and HPC environments enable large-scale processing, and miniaturized, intelligent sensor systems are expanding data acquisition capabilities. These innovations are creating comprehensive “space–air–ground” monitoring networks that combine satellite, aerial and ground-based observations.

Targeted advancements are being seen in the following application areas:

- Agriculture: Dual-purpose UAVs and thermal imaging for precision farming
- Disaster management: Integrated systems for early landslide detection
- Urban planning: Global air quality monitoring through multi-sensor fusion
- Ecology: Biodiversity conservation via interdisciplinary data integration

The field is moving towards web-based spatiotemporal analysis systems and enhanced visualization tools, supported by machine learning techniques such as SVM and ensemble methods. These developments are fostering new collaborations between remote sensing experts and specialists in ecology, climate science and urban planning.

This technological convergence is creating more robust environmental monitoring frameworks, enabling timely decision-making and sustainable resource management at all scales. Integrating diverse data streams and analytical methods promises to provide us with unprecedented insights into the processes of the Earth system and the interactions between humans and the environment [56,58,125,126].

## 6. Conclusions

This review has summarized advancements in land monitoring, highlighting the critical need for and inherent advantages of integrating data from optical, radar, and LiDAR satellite sensors. The synergistic harmonization of these distinct remote sensing modalities provides a more comprehensive and robust understanding of land surface characteristics and dynamics than relying on single sensor types alone. Optical imagery provides rich spectral information, radar offers all-weather, day-and-night capabilities sensitive to structure and dielectric properties, while LiDAR excels in delivering precise three-dimensional structural data. The combination of these data streams addresses the limitations inherent in single-source approaches, such as susceptibility to atmospheric conditions or insufficient information depth [43,74,114,127,128,129,130,131].

Significant progress has been made in methodologies for integrating these diverse data sources. Machine learning, particularly deep learning, has demonstrated substantial promise and achieved notable success in multi-modal remote sensing data fusion. Techniques like multi-attentive hierarchical fusion have shown improved land cover classification accuracy by integrating modality-specific and combined features, as demonstrated in the fusion of hyperspectral and LiDAR data. AI-driven approaches, including those combining artificial intelligence with human expertise, are crucial for advancing land cover mapping accuracy and efficiency. Furthermore, cloud-based platforms and scalable analytics have emerged to handle the computational challenges associated with large-scale Earth observation data, enabling continental-scale land cover change analysis over extended periods. Openly available datasets, such as GLC30 and GLC_FCS30D, derived from multi-source optical data and leveraging machine learning, underscore the increasing accuracy and potential for detailed, multi-temporal land cover monitoring at global and regional scales [90,129,132,133,134,135].

Despite the significant advancements, several challenges persist. The limitations of single remote sensing technologies in areas such as anti-interference, all-weather capability, imaging noise, richness of classification information, and data coverage integrity necessitate integrated approaches. Obtaining consistently high-quality monitoring data that meets specific application requirements remains difficult. Furthermore, the generalization ability of existing hazard monitoring methods can be limited, and the complexity of dynamic processes, such as geohazard evolution, requires dynamic monitoring beyond periodic observations to avoid lagging risk management. Early identification of undetectable geohazards, for instance, remains a critical challenge. Collaboration among stakeholders is also essential for strategically developing satellite capabilities and optimizing resource allocation [111,136,137,138].

Future research directions should focus on addressing these challenges through continued innovation in data fusion techniques, particularly multi-level fusion strategies for multi-source data. Developing more accurate and generalizable identification and monitoring models, including those guided by domain-specific knowledge, is crucial. Advancements in deep learning models and data fusion approaches, including the exploration of visual-language models in remote sensing, offer promising avenues. Enhanced international collaboration and the development of new technologies for processing multi-temporal imagery and modeling long-term geo-processes will further stimulate progress. Continued development and in-depth information mining from LiDAR technology will also promote its applications in three-dimensional natural resource monitoring [139].

The synergistic integration of optical, radar, and LiDAR satellite data, powered by advanced analytics and artificial intelligence, offers unprecedented potential for comprehensive land monitoring. This capability is indispensable for providing critical, timely, and accurate information essential for informed decision-making across a spectrum of vital areas. These include environmental management, urban planning, disaster response (such as wildfire monitoring, flood mapping, landslide and geohazard detection), sustainable natural resource management (water, forests, land), agricultural resource management, and understanding land use dynamics related to urbanization, agricultural intensification, and climate change. Synergistic land monitoring has become a core tool for addressing environmental degradation, resource conflicts, and climate crises through technological integration (EO + ML), institutional integration (LAS interdepartmental collaboration), and policy coordination (SDGs indicator embedding). Ultimately, advancing synergistic land monitoring is fundamental to addressing global environmental and societal challenges and supporting the sustainable development goals.

## Figures and Tables

**Table 1:** Sensor type and performances.

Sensor Type	Key Strengths	Key Weaknesses	Key Information Provided	Example Platforms
Optical	Rich spectral and textural info; high spatial resolution (panchromatic)	Limited by clouds; solar illumination required	Spectral reflectance, color, texture, vegetation indices (NDVI)	Landsat; Sentinel-2; Gaofen
Radar (SAR)	All-weather; day/night, penetration; sensitivity to structure/moisture	Speckle noise; less intuitive visualization; phase decorrelation (InSAR)	Backscatter (roughness, dielectric); deformation (InSAR); urban structure	Sentinel-1; RADARSAT-2; Gaofen-3
LiDAR	Precise 3D structure; canopy penetration; high-accuracy elevation	High cost; limited swath/coverage; sensitivity to atmospheric particles; limited spectral info	elevation (DEM, DTM); canopy height (CHM); Point cloud density; intensity	ICESat-2; GEDI, GaoFen-7; Airborne/UAV

**Table 2:** Fusion level and parameters.

Fusion Level	Description	Input	Output	Key Characteristics/Advantages	Challenges/Considerations
Pixel-level	Directly combine raw pixel values	Raw multi-sensor images	Fused image (enhanced spatial/spectral resolution)	Enhance spatial/spectral detail; integrate different data types	Requires precise geometric alignment; sensitivity to scale differences
Feature-level	Extract features from each source, then combine features	Extracted features (indices, texture, etc.)	Combined feature vector/map	Reduces data dimensionality; handles multi-band data; tailored features	Feature selection complexity; potential redundancy; still requires pixel registration
Decision-level	Process each source independently, then combine results/decisions	Decisions/results from independent analysis	Final combined decision	Useful for highly heterogeneous data; independent processing pipelines	Handling conflicting information; difficulty resolving disagreements

**Table 3:** Challenges of synergistic harmonization.

Category	Key Challenges
Technical	Integrating diverse data (spectral, spatial, temporal, noise); geometric and radiometric issues (co-registration, pan-sharpening, speckle); computational demands (large data); algorithm generalization; handling missing data.
Data Availability and Accessibility	Scarcity of large, open datasets (esp. SAR for AI); limited diversity of change types in datasets; high cost/effort of dataset creation/maintenance; real-time data limitations.
Validation and Accuracy Assessment	Scarcity of high-quality ground truth data; validation across diverse environments; reliance on labeled data (for DL).
Standardization	Lack of robust guidelines, protocols, and benchmarks for multi-sensor fusion, processing, and assessment.

**Table 4:** Advancements/opportunities of synergistic harmonization.

Category	Advancements/Opportunity
AI and ML	Widespread application of deep learning (CNNs, RNNs, GANs, Transformers); foundation models; vision-language models; human–machine synergy.
Data Fusion Techniques	Moving beyond traditional methods (fuzzy data, reliability estimates); multi-level fusion strategies; unified DL frameworks; hardware fusion systems.
Data Access and Infrastructure	Cloud computing and big data analytics; miniaturization, integration, and intelligence of sensors; new satellite missions (CO3D, Pléiades Neo Next, etc.).
System Integration	Integrated “space–air–ground” monitoring systems; combining satellite data with UAVs, ground sensors, and GNSS.
Application-Specific Advancements	Improved models for specific tasks (damage assessment, geohazards, urban, agriculture); high-resolution mapping; global product generation; spatiotemporal analysis.
Interdisciplinary Integration	Combining RS data with ecological, evolutionary, socio-economic, weather, and other allied data for holistic analysis.

## Data Availability

Data are contained within the article.
